# Characterization and Antimicrobial Activity of a Halophyte from the Asturian Coast (Spain): *Limonium binervosum* (G.E.Sm.) C.E.Salmon

**DOI:** 10.3390/plants10091852

**Published:** 2021-09-07

**Authors:** Eva Sánchez-Hernández, Laura Buzón-Durán, Natalia Langa-Lomba, José Casanova-Gascón, Belén Lorenzo-Vidal, Jesús Martín-Gil, Pablo Martín-Ramos

**Affiliations:** 1 Agriculture and Forestry Engineering Department, ETSIIAA, Universidad de Valladolid, Avenida de Madrid 44, 34004 Palencia, Spain; eva.sanchez.hernandez@uva.es (E.S.-H.); laura.buzon@uva.es (L.B.-D.); mgil@iaf.uva.es (J.M.-G.); 2 Instituto Universitario de Investigación en Ciencias Ambientales de Aragón (IUCA), EPS, Universidad de Zaragoza, Carretera de Cuarte, s/n, 22071 Huesca, Spain; natalialangalomba@gmail.com (N.L.-L.); jcasan@unizar.es (J.C.-G.); 3 Servicio de Microbiología, Hospital Universitario Rio Hortega, Calle Dulzaina 2, 47012 Valladolid, Spain; blorenzov@saludcastillayleon.es

**Keywords:** antibacterial, antifungal, *Diplodia seriata*, *Erwinia amylovora*, rock sea lavender, *Xylophilus ampelinus*

## Abstract

The work presented herein deals with the characterization and valorization of a halophyte from the cliffs of the Asturian coast: *Limonium binervosum* (G.E.Sm.) C.E.Salmon (rock sea-lavender). Its biomass and hydromethanolic extracts were studied by elemental and thermal analysis, infrared spectroscopy and gas chromatography–mass spectroscopy. Tetradecanoic acid/esters and 1,2-tetradecanediol were identified in its flower extract, while the leaf extract was rich in linolenic and linoleic acids and their esters, hexadecanoic acid and its esters, and phytol. Both flower and leaf hydromethanolic extracts contained eicosane, sitosterol and tocopherols in significant amounts. With a view to its valorization, the antimicrobial activity of these extracts was investigated against three apple tree and grapevine phytopathogens. Both the hydroalcoholic extracts and their main constituents, alone or in combination with chitosan oligomers (COS), were tested in vitro. A remarkable antibacterial activity was observed for the conjugated complexes of the flower extract with COS, both against *Xylophilus ampelinus* (MIC = 250 μg·mL^−1^) and *Erwinia amylovora* (MIC = 500 μg·mL^−1^), and complete inhibition of the mycelial growth of *Diplodia seriata* was found at concentrations <1000 μg·mL^−1^. In view of these results, this extremophile plant can be put forward as a promising source of bioactive metabolites.

## 1. Introduction

*Limonium* is one of the most important species-rich genera in the *Plumbaginaceae* family. This widespread genus of halophytes and taxa includes sexual diploids of the *L. ovalifolium* (Poir.) Kuntze complex, the triploid *L. algarvense* Erben and the agamospermous tetraploids of the *L. binervosum* (G.E.Sm.) C.E.Salmon complex [1]. The *L. binervosum* aggregate is a species group that has not been assigned to any of the subsections of *L.* sect. *Limonium* [2] and was first reported in 1922 by Salmon [3].

**Figure 1:**
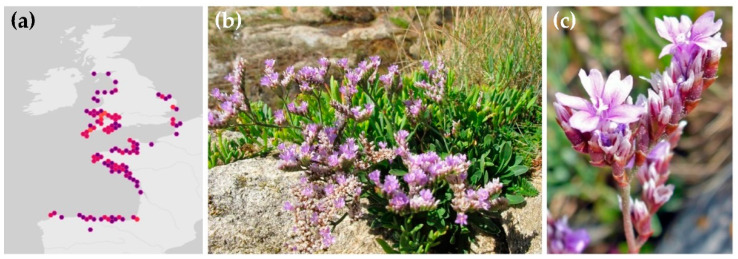
(**a**) habitat of *Limonium binervosum* (G.E.Sm.) C.E.Salmon; *L. binervosum* in cliffs in Llanes (Asturias, Spain): (**b**) whole plant and (**c**) flowers. Credit: habitat map generated with OpenStreetMap using GBIF data, under CC BY-NC license.

The habitat of *L. binervosum* includes coastal cliffs, pebble beach margins, steppes, meadows and lagoons. It grows on the Atlantic coasts of Europe, from the south-west United Kingdom and north-west France to northern Spain, with a number of geographically restricted segregate taxa (Figure 1a).

Plants of *L. binervosum* can grow up to 20 cm, and have regular, straight spikes, which are not aggregated into a corymbose head (Figure 1b). Stems divide several times. Leaves are narrow oblanceolate, greyish-green in color, with a midvein. The flowers form in compact clusters along the leafless stem branches and are pink, formed of five notched petals, with five short stamens topped by white anthers and a purplish calyx (Figure 1c) [4].

A review of the bioactive components in several species of the *Limonium* genus suggests that they are a good source of antioxidants. For instance, in *L. algarvense* flowers, the antioxidants are related to gallic acid, catechin, salicylic and rosmarinic acids, and epigallocatechin gallate [5], similar to those found in *L. brasiliense* (Boiss.) Kuntze (viz. gallic acid, gallocatechin, epigallocatechin, PDE gallate, etc.) [6]. In *L. aureum* (L.) Hill, the antioxidants identified were myricetin (or cannabiscetine), myricetin-3-*O*-glucoside, myricetin-3-*O-β*-D-glucopyranoside, myricitrin, erioictyol, homoeridictyol, and eriodictyol-7-O-glucoside [7]. In a study on the leaves of *L. delicatulum* (Girard) Kuntze and *L. quesadense* Erben it was found that the former is rich in myricetin glycosides, whereas in the latter epigallocatechin gallate and its dimer are some of the most abundant compounds [8]. Consequently, *L. binervosum*—whose phytochemical constituents have not been studied to date—may also be a promising source of antioxidants.

Taking into consideration that antioxidant activity is generally associated with antibacterial, antifungal and antimycotoxigenic biological activities [9], potential valorization strategies for *L. binervosum* as a source of bioactive products may be envisaged, aligned with the premises of current EU regulation (Directive 2009/128/EC on the sustainable use of pesticides, Council Regulation (EC) 834/2007 on organic production and labeling of organic products, Regulation (EU) 2019/1009 on the market of EU fertilizing products, etc.), in which the replacement of conventional phytosanitary products with formulations based on natural products is encouraged.

In particular, in this study, its application to the control of grapevine (*Vitis vinifera* L.) and apple tree (*Malus domestica* Borkh.) pathogens was explored by assessing its antibacterial activity against *Xylophilus ampelinus* (Panagopoulos 1969) Willems et al. 1987 and *Erwinia amylovora* (Burrill), and its antifungal activity against *Diplodia seriata* De Not.

*X. ampelinus* (syn. *Xanthomonas ampelina* and *Erwinia vitivora* [10]), a quarantine A2 organism according to the European and Mediterranean Plant Protection Organization (EPPO), causes the bacterial necrosis of grapevines (“mal nero” or “maladie d’Oléron”), resulting in yield losses of up to 70% [11]. *E. amylovora*, also cataloged as a quarantine organism, causes fire blight, which poses a serious threat to pear and apple production [12]. In turn, *D. seriata*, a Botryosphaeriaceous fungus, causes dieback, canker, leaf spot and fruit rot in a wide range of hosts, including grapevine [13,14] and apple trees [15,16,17].

With a view to a possible valorization of this halophyte (*L. binervosum*), a physicochemical characterization is presented, together with an in vitro evaluation of the antimicrobial activity of its extracts—alone and in combination with chitosan oligomers—against aforementioned phytopathogens.

## 2. Results

### 2.1. Elemental Analysis and Calorific Values Calculation

The C, H, N and S percentages of *L. binervosum* components (wt% of dry material) were in the 40.5–44.7%, 6.4–6.5%, 1.2–2.6% and 0.2–0.9% range, respectively (Table 1). 

Higher heating values derived from elemental analysis data resulted in heating values for flowers and leaves of 18 and 16 kJ·g^−1^, respectively.

### 2.2. Thermal Analyses

The TG, DTG and DSC curves of flowers and leaves are shown in Figures S1 and S2, respectively. In the case of flowers, exothermal effects were detected at 329, 420 and 470 °C; the ash content (at 550 °C) was 5.6%. Concerning leaves, exothermal effects were registered at 320 and 470 °C, and the ash content (at 580 °C) reached 17%. For comparison purposes, the total ash content reported *L. stocksii* (Boiss.) Kuntze was 11.83% [18].

### 2.3. Vibrational Characterization

The main absorption bands in the FTIR spectra of the powdered dry samples of flowers and leaves are summarized in Table 2, together with their assignments. The bands at 2918, 2850, 1462 and 720 cm^−1^ are due to aliphatic features and are present in straight-chain alkanes (compatible with the presence of tetracosane, pentacosane, heptacosane, etc., identified by GC–MS in the extracts, as discussed below) [19]. The band at 2158 cm^−1^, ascribed to C-N stretching, may arise from the presence of carbonitrogenated compounds (e.g., n-methyl-1-adamantaneacetamide; 2-(4-fluoro-phenyl)-4-(3-methyl-benzylidene)-4h-oxazol-5-one, 2-ethylacridine, etc.) [20]. The bands at ca. 1730 and ca. 1165 cm^−1^, related to carbonyl (C=O) stretching and C-C(=O)-O stretching, respectively, illustrate the main spectral features of esters (e.g., 2-hydroxy-tetradecanoic acid methyl ester; hexadecanoic acid methyl ester; 9,12-octadecadienoic acid methyl ester; 9,12,15-octadecatrienoic acid methyl ester, etc.) [19]. The band at ca. 1640 cm^−1^, resulting from C=O and C=C stretching vibrations and asymmetric N–H bending vibrations, can be due to flavonoids and lipids [21,22]. The bands at 1513 and 1417 cm^−1^, related to aromatic C=C stretching, are compatible with the presence of flavonoids and aromatic rings. The band at 1235 cm^−1^ may be due to C–O group vibration in polyols, such as hydroxyflavonoids [23].

The FTIR spectrum of the lyophilized flower hydromethanolic extract (not included in Table 2) showed bands at 3362, 2917, 2849, 1733sh, 1636, 1462, 1340, 1228, 1067 and 957 cm^−1^, attributable to tetradecanoic (1727, 1448, 1310 cm^−1^) and eicosane (2914, 2847 and 1471 cm^−1^). 

### 2.4. Hydromethanolic Extracts Characterization

#### 2.4.1. Phenolic Contents

The total phenolic content of the flower and leaf extracts were 162 ± 7 and 58 ± 2 mg GAE/g DW, respectively.

#### 2.4.2. Analysis of Hydromethanolic Extracts by GC–MS

The main constituents identified in the flower hydromethanolic extract (Table 3 and Table S1, and Figure S3) were: tetradecanoic acid and methyl 2-hydroxy tetradecanoate (22%); eicosane (18%); 1,2-tetradecanediol (15%); sitosterol (9%); tocopherols/vitamin E (7%); and *n*-alcanes (heneicosane, tetracosane, pentacosane, heptacosane, etc., which add up to 6%). Among the minority constituents, it is necessary to highlight the presence of 2-ethyl-acridine (1.6%) as the only carbonitrogenated compound. 

Concerning the main phytoconstituents identified in the leaf extract (Table 4 and Table S2, and Figure S4), they were: octadecatrienoic acid (linolenic acid) and their esters (above 22%); sitosterol (19%); hexadecanoic acid and their esters (above 15%); octadecadienoic acid or linoleic acid (8%); vitamin E (8%) and other tocopherols (5%); trans-pinane (5%); eicosane (4%); and phytol (4%).

### 2.5. Antimicrobial Activity

#### 2.5.1. In Vitro Antibacterial Activity

The inhibition of flower and leaf extracts against *X. ampelinus* and *E. amylovora* were similar and comparable to that attained with COS (Table 5). Regarding the activities of the main active principles present in the extracts, differences were observed as a function of the pathogen: while tetradecanoic acid, linolenic acid and vitamin E showed similar activity against *X. ampelinus* (MIC = 500 μg·mL^−1^), tetradecanoic acid was the most effective against *E. amylovora* (MIC = 500 μg·mL^−1^), and linolenic acid and vitamin E were less effective (MIC = 750 μg·mL^−1^). β-sitosterol showed worse performance than the former three (MIC = 1000 and 1500 μg·mL^−1^ against *X. ampelinus* and *E. amylovora*, respectively), and eicosane was the least effective (MIC = 1000 and >1500 μg·mL^−1^ against *X. ampelinus* and *E. amylovora*, respectively).

Upon conjugation with COS, a synergistic behavior was observed for all phytochemicals. The best results against *X. ampelinus* were attained with the COS–flower extract conjugate complex (MIC = 250 μg·mL^−1^), comparable to those attained for the COS–tetradecanoic acid, COS–linolenic acid and COS–vitamin E conjugate complexes, while the effectiveness of the COS–leaf extract was lower (MIC = 500 μg·mL^−1^). In the case of *E. amylovora*, the COS–flower extract conjugate complex was more effective than the leaf-based one (MIC = 500 μg·mL^−1^ vs. 750 μg·mL^−1^, respectively), but less effective than the COS–tetradecanoic acid, COS–linolenic acid and COS–vitamin E conjugate complexes (MIC = 250 μg·mL^−1^, similar to those observed against *X. ampelinus*).

#### 2.5.2. In Vitro Antifungal Activity

**Figure 2:**
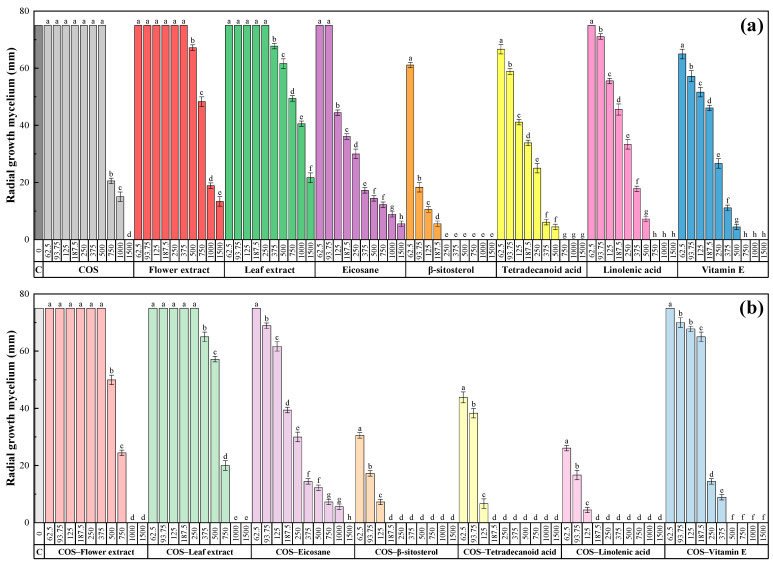
Radial growth of the mycelium for *D. seriata* in in vitro tests conducted in PDA medium with different concentrations (62.5, 93.75, 125, 187.5, 250, 375, 500, 750, 1000 and 1500 µg·mL^−1^) of chitosan oligomers (COS), *L. binervosum* flower and leaf extracts, and their main phytochemical constituents (**a**), and their respective conjugate complexes (**b**). The same letters above concentrations mean that they are not significantly different at *p* < 0.05. Error bars represent standard deviations.

The results from the *D. seriata* mycelial growth inhibition tests are shown in Figure 2 and Figure S5. At the highest dose (1500 μg·mL^−1^), the flower and the leaf extracts resulted in 82% and 71% inhibition, respectively, while full inhibition was attained at 750 μg·mL^−1^ for tetradecanoic acid, linolenic acid and vitamin E constituents, and at 250 μg·mL^−1^ for β-sitosterol. In the case of eicosane, 93% inhibition was observed at the highest dose.

The formation of conjugate complexes improved the activity in all cases, with remarkable improvements in COS–tetradecanoic and COS–linolenic (from 750 down to 187.5 μg·mL^−1^). Concerning flower and leaf extracts, full inhibition was attained at 1000 μg·mL^−1^ in both cases.

Determination of EC_50_ and EC_90_ values (50% and 90% maximal effective concentration, respectively), summarized in Table 6, and calculation of synergy factors, presented in Table 7, confirmed the strong synergistic behavior previously mentioned for COS and tetradecanoic and linolenic acids (with SFs of 4.55 and 5.75 for the EC_90_, respectively). In all the other cases, SFs > 1 (i.e., indicative of a synergistic behavior) were also obtained, albeit more moderate.

## 3. Discussion

### 3.1. Elemental Analysis and Calorific Values Calculation

In relation to the elemental analysis results, the carbon content is close to that reported by Park et al. [24] for *L. tetragonum* (Thunb.) Bullock (45.5%), while the nitrogen content in leaves is in good agreement with that reported for *L. echioides* (L.) Mill. (ca. 2.4%) for complete shoots [25]. The fact that the values of the C/N ratios for flowers are twice those obtained for leaves is consistent with the higher percentage of carbonitrogenated compounds in leaves (viz. *n*-methyl-1-adamantaneacetamide, and 2-(4-fluoro-phenyl)-4-(3-methyl-benzylidene)-4h-oxazol-5-one, which account for ca. 3% according to GC–MS results) than in flowers (viz. 2-ethylacridine, 1.59%).

The calorific values obtained from elemental analysis data, below the 18.82 kJ·g^−1^ limit required in EN 14961-2 [26], and the high ash contents (above the 2% limit), preclude the valorization of this halophyte as solid biofuel. Nonetheless, it is worth noting that the fatty acid profile (discussed below), rich in linolenic and linoleic acids, can make *L. binervosum* a promising biofuel feedstock, according to Patel et al. [27].

### 3.2. Phytochemical Composition

The eicosane content in the flower extract (18%) is higher than the one reported in the aerial parts of *L. leptophyllum* (Schrenk) Kuntze (8%) [28]. Concerning *β*-sitosterol, its presence was reported in the rhizome of *L. brasiliense* [6], *L. myrianthum* (Schrenk) Kuntze [28], *L. gmelinii* (Willd.) Kuntze and *L. popovii* Kubansk. [29] and in the aerial parts of *L. axillare* (Forssk.) Kuntze [30]. Tetradecanoic, linolenic and linoleic acids were reported in the aerial parts and roots of *L. gmelinii* and *L. popovii* [29], with contents in the 1–4%, 11–27% and 15–32% range, respectively (vs. 22%, 22% and 8%, respectively, for *L. binervosum*).

Although flavonol myricetin (3,5,7-trihydroxy-2-(3,4,5-trihydroxyphenyl)-4-chromenone), reported for *L. aureum* [7] and *L. delicatulum* [8], was not found among the phytochemicals identified by GC–MS in our experimental conditions, significant amounts (7–13%) of antioxidants alternative to myricetin, such as the *α*-, *β*-, *γ*- and *δ*-tocopherols (e.g., 2-[(4R,8R)-4,8,12-trimethyltridecyl]-3,4-dihydrochromen-6-ol natural vitamin E constituents) were identified. This feature is important because in the literature [31,32] some antimicrobial activity was advocated for myricetin analogs, and a synergistic antioxidant effect of *α*-tocopherol and myricetin was described [33].

Concerning the TPC of the flower extract (162 mg GAE/g DW), it was higher than those reported for *L. sinuatum* (L.) Mill. flowers (23–34 mg GAE/g DW) [34,35], but lower than those reported for *L. algarvense* flower methanol extract (228 mg GAE/g DW) [5]. In regards to the TPC in the leaf extract (58 mg GAE/g DW), it was similar to those reported for *L. delicatulum* shoot extracts (47 mg GAE/g DW) [36]: *L. densiflorum* (Guss.) Kuntze shoots (50–56 mg GAE/g DW) [37,38], *L. algarvense* leaves (54 mg GAE/g DW) [5], and *L. morisianum* Arrigoni aerial parts (59 mg GAE/g DW) [39]. These values are in the lower end of the range reported by Senizza et al. [40] and Ruiz-Riaguas et al. [8] for *L. delicatulum*, *L. quesadense, L. bellidifolium* (Gouan) Dumort., *L. globuliferum* (Boiss. and Heldr.) Kuntze, *L. gmelinii*, *L. iconicum* (Boiss. and Heldr.) Kuntze, *L. lilacinum* (Boiss. and Balansa) Wagenitz and *L. sinuatum* aerial parts extracts (44–172 mg GAE/g DW).

### 3.3. Antimicrobial Activity of Limonium spp. Extracts

The use of halophytes to obtain bioactive antimicrobial extracts is recent, and the effect of the natural products derived from them was generally evaluated against human pathogens (as in the case of the extracts from *Pistacia atlantica* Desf., *Tamarix gallica* L., *T. articulata* Vahl, *Anabasis articulata* (Forssk.) Moq. or *Suaeda fructicosa* (L.) Forssk. [41,42,43,44]), not against phytopathogens.

In the particular case of *Limonium* genus., antimicrobial studies were reported for other species, such as *L. brasiliense* [6], *L. awei* (De Not.) Brullo and Erben [45,46], *L. morisianum* [39], *L. socotranum* (Vierh.) Radcl.-Sm. [47], *L. echioides* [48], *L. densiflorum* [37], *L. delicatulum* [36], *L. myrianthum, L. leptophyllum* and *L. gmelinii* [49], but not for *L*. *binervosum*, so direct efficacy comparisons are not possible.

Regarding the antibacterial activity, Blainski et al. [6] reported a desirable inhibition of bacterial growth for the ethyl-acetate fraction of ternary extracts of *L. brasiliense* against vancomycin-resistant *Enterococcus faecium*, methicillin-resistant *Staphylococcus aureus* and *Klebsiella pneumoniae*, with MIC values of 19, 39 and 625 μg·mL^−1^, respectively. The activity of *L. awei* extracts was reported by Filocamo et al. [45], with MIC and minimum bactericidal concentration (MBC) values ranging from 15.6 to 500 μg·mL^−1^ and from 500 to 4000 μg·mL^−1^, respectively, against Gram-positive bacteria and >2000 μg·mL^−1^ for Gram-negative bacteria. For the same *Limonium* species, Nostro et al. [46] reported MIC and MBC values ranging from 7.8 to 62.5 μg·mL^−1^ and from 500 to 2000 μg·mL^−1^, respectively, against *S. aureus* (including methicillin-resistant strains). Recently, Mandrone et al. [39] found potent anti-staphylococcal properties for *L. morisianum* extract, with an average IC_50_ value of 9.2 [6.8–12.3] μg·mL^−1^. Moreover, recently, Al-Madhagi et al. [47] noted that methanol leaf and flower extracts from *L. socotranum* exhibited higher antibacterial activity against *Micrococcus luteus* (MIC 15.6 μg·mL^−1^), *S. aureus* (MIC 125 μg·mL^−1^) and *Pseudomonas aeruginosa* (MIC 125 μg·mL^−1^) than stem extracts.

Concerning the antifungal activity of *Limonium* spp., a low antifungal activity was reported for *L. echioides* (against *Fusarium oxysporum* and *Penicillium* sp. [48]), for *L. avei* (against *Candida albicans* [46]), and for *L. densiflorum* and *L. delicatilum* (against *Candida* spp. [36,37]). Nonetheless, a stronger antifungal activity against *C. albicans* and *Aspergillus niger*, with full inhibition at concentrations as low as 62 and 125 μg·mL^−1^, respectively, were found for *L. socotranum* leaf and flower extracts [47]. Significant antifungal activities against *C. glabrata*, with IC_50_ values in the 4.96–6.83 μg·mL^−1^, were also reported for secondary metabolites from *L. myrianthum, L. leptophyllum* and *L. gmelinii* by Gadetskaya et al. [49].

### 3.4. Antimicrobial Activity of the Main Identified Phytochemicals

All the main phytochemicals found in the *L. binervosum* flower and leaf extracts have been reported to have both antimicrobial and antifungal activity (albeit not against any of the phytopathogens referred herein).

Eicosane is effective against bacteria such as *Escherichia coli*, *Salmonella typhi* and *S. aureus* [50], and against fungi such as *Rhizoctonia solani* [51]. Likewise, the antimicrobial activity of *β*-sitosterol against both bacteria (*S. typhii, Corynebacterium diphtheriae, Bacillus subtilis, Shigella dysenteriae* and *Vibrio cholerae*) and fungi (*Fusarium* spp. and *Penicillium* spp.) was reported by Kiprono et al. [52].

Concerning fatty acids, which are the major constituents of *L. binervosum* extracts, it was demonstrated that the antibacterial action of long-chain unsaturated fatty acids is mediated by the inhibition of fatty acid synthesis [53], and it was shown that both saturated and unsaturated fatty acids have antifungal activity, although saturated fatty acids would show a stronger activity [54]. In particular, antimicrobial properties of tetradecanoic acid were referred to in the literature (against, for instance, *Listeria monocytogenes* [55] and *C. albicans* [56]), as well as for its derivatives, such as methyl 2-hydroxytetradecanoate (against *C. albicans*, *Cryptococcus neoformans* and *A. niger* [57]). Regarding linolenic acid, Lee et al. [58] concluded that this fatty acid has a strong antibacterial activity against *B. cereus* and *S. aureus*, and Walters et al. [59] showed its activity against *R. solani*, *Pythium ultimum*, *Pyrenophora avenae* and *Crinipellis perniciosa*.

With regard to vitamin E, its antibacterial activity against *E. coli, S. aureus, S. epidermidis, P. aeruginosa*, *Proteus* spp., *Klebsiella* spp., and *Enterobacter* spp. was evidenced by Al-Salih et al. [60], and it was reported that—in combination with fluconazole—it results effective in the treatment of some human fungal diseases [61].

### 3.5. On the Synergistic Behavior Observed for the Conjugate Complexes

The combination of chitosan with several of the main constituents of *L. binervosum* extracts has precedents in the literature. For instance, combinations of chitosan with vitamin E were studied by Yeamsuksawat and Liang [62], Martins et al. [63] and Raza et al. [64]. The rationale behind such choice is that, while *α*-tocopherol has feeble stability, it is improved by encapsulation in chitosan as a capping agent, as well as its release when required over a sustained period. Similarly, Liu et al. [65] reported the formation of self-assembled nanoparticles by coupling chitosan with linolenic acid, taking advantage of the fact that chitosan is known to inhibit the linoleic (and linolenic) acid oxidation process [66]. In the case of tetradecanoic acid, chitosan–tetradecanoic acid nanogels with MIC values of 10 mg·mL^−1^ against *S. enterica* were reported by Rajaei et al. [67].

Nonetheless, none of the aforementioned combinations are conjugated complexes, and the existence of interactions between the two components in terms of antimicrobial activity was not explored. Albeit for other phytochemicals different from the ones present in *L. binervosum*, a synergistic behavior upon conjugation with COS was reported in the literature against phytopathogens: e.g., for horsetail (*Equisetum arvense* L.) and nettle (*Urtica dioica* L.) extracts against eight fungal species involved in grapevine trunk diseases [68], with EC_90_ values in the 208–1000 μg·mL^−1^ range (depending on the extract and on the *Botryosphaeriaceae* taxa). The value reported in this work for the COS–flower extract complex (914 μg·mL^−1^) would be on the upper limit.

For the same phytopathogens studied herein, and also for extracts from halophytes, MIC values of 375 and 500 μg·mL^−1^ against *X. ampelinus* and 187.5 and 500 μg·mL^−1^ against *E. amylovora* were found for the conjugate complexes formed between COS and rock samphire (*Crithmum maritimum* L.) and sea carrot (*Daucus carota* subsp. *gummifer* (Syme) Hook.fil.) hydromethanolic extracts, respectively. Such inhibition values are worse than the one reported herein against *X. ampelinus* for the COS–flower extract conjugate complex (MIC = 250 μg·mL^−1^), but slightly better than/comparable to that obtained against *E. amylovora* (MIC = 500 μg·mL^−1^) [69].

The mechanism of synergistic action of such COS-phytochemical conjugates has not been dilucidated yet. Nonetheless, it was suggested that it might be the result of an enhanced additive antimicrobial effect, per se, and/or via a concurrent action on diverse microbial metabolic sites. An increase in the cationic surface charge of COS may also result from conjugation with phytochemicals, which would enhance the linkage to negatively charged site-specific binding receptors on the bacterial/fungal membranes [70,71,72,73].

## 4. Material and Methods

### 4.1. Reagents

High-molecular weight (310,000–375,000 Da) chitosan (CAS 9012-76-4) was purchased from Hangzhou Simit Chem. and Tech. Co. (Hangzhou, China). Neutrase^TM^ 0.8 L enzyme was obtained from Novozymes A/S (Bagsværd, Denmark). The preparation of chitosan oligomers (COS) was carried out according to the procedure reported by Santos-Moriano et al. [74], with the modifications indicated in [73].

Eicosane (CAS 112-95-8, 99%), 1,2-tetradecanoic acid (CAS 544-63-8, Sigma Grade, ≥99%), linolenic acid (CAS 463-40-1, ≥99%), *β*-sitosterol (CAS 83-46-5, analytical standard), vitamin E (*α*-tocopherol, CAS 10191-41-0, analytical standard), methanol (CAS 67-56-1, UHPLC, suitable for MS), tryptic soy broth (TSB, CAS 8013-01-2) and tryptic soy agar (TSA, CAS 91079-40-2) were supplied by Sigma-Aldrich (Madrid, Spain). Potato dextrose agar (PDA) was acquired from Becton Dickinson (Bergen County, NJ, USA). All reagents were used as supplied without further purification.

### 4.2. Plant Material and Extraction Procedure

*L. binervosum* was collected in sea cliffs in Llanes (Asturias, Spain; 43°26′10.7″ N 4°49′25.1″ W) in early September 2020. Separate composite samples of flowers and leaves were obtained by thoroughly mixing the aerial parts from different specimens (*n* = 15). The composite samples were shade-dried, pulverized in a mechanical grinder, homogenized and sieved (1 mm mesh).

*L. binervosum* flower samples were mixed (1:20 *w*/*v*) with a 1:1 *v*/*v* methanol:water solution and heated for 30 min in a water bath at 50 °C. Subsequently, they were sonicated for 5 min in pulse mode with a 1 min stop for each 2.5 min, using a Hielscher Ultrasonics (Teltow, Germany) probe-type ultrasonicator (model UIP1000hdT; 1000 W, 20 kHz). The solution was subjected to centrifugation at 9000 rpm for 15 min, and the supernatant was filtered through Whatman No. 1 paper. Aliquots were lyophilized for CHNS and FTIR analyses. The extraction procedure for leaf samples was identical.

Each extraction procedure was replicated three times (on subsamples of the flower and leaf composite samples), and the resulting hydromethanolic extracts were mixed to obtain the samples for GC‒MS analysis.

### 4.3. Bacterial and Fungal Isolates

The *X. ampelinus* and *E. amylovora* bacterial isolates were supplied by CECT (Valencia, Spain), with CCUG 21976 and NCPPB 595 strain designations, respectively. *D. seriata* (isolate Y-084-01-01a, code ITACYL_F098), obtained from “Tempranillo” grapevine plants from P.D.O. Toro (Spain), was supplied by ITACYL (Valladolid, Spain) [75] as a lyophilized vial, which was reconstituted and refreshed as a PDA subculture.

### 4.4. Physicochemical Characterization

Elemental analyses of dry ground samples (3 mg/sample) were performed with a CHNS-932 apparatus (LECO, St. Joseph, MI, USA).

Calorific values were calculated from elemental analysis data according to Talwalkar et al. [76], using the following equation:

(1) HHV=(0.341 × %C)+(1.322 × %H) − 0.12 (%O+%N),

where HHV is the heating value for the dry material, expressed in kJ·g^−1^, and %C, %H, %O and %N are the mass fractions, expressed in wt% of dry material.

Thermal gravimetric (TGA) and differential scanning calorimetry (DSC) analyses were conducted with a simultaneous TG-DSC2 apparatus (Mettler Toledo; Columbus, OH, USA). Samples (10 mg/sample) were heated from 30 to 600 °C under N_2_:O_2_ (4:1) flow (20 cm^3^·min^−1^), at a 20 °C·min^−1^ heating rate.

The infrared spectra were obtained with a Nicolet iS50 Fourier-transform infrared spectrometer (Thermo Scientific; Waltham, MA, USA), equipped with an in-built diamond attenuated total reflection (ATR) system. A spectral resolution of 1 cm^−1^ over the 400–4000 cm^−1^ range was used, taking the interferograms that resulted from co-adding 64 scans.

The colorimetric quantification of the total polyphenol content (TPC), expressed in gallic acid equivalents (GAE), was conducted according to the procedure described in [77], using a UV-Vis Cary 100 spectrometer (Agilent Technologies; Santa Clara, CA, USA).

The gas chromatography–mass spectrometry (GC–MS) analyses of the hydroalcoholic plant extracts (obtained as a mixture of three extractions) were carried out at the Research Support Services (STI) at Universidad de Alicante (Alicante, Spain). A model 7890A gas chromatograph coupled to a model 5975C quadrupole mass spectrometer (Agilent Technologies). The chromatographic conditions were: 3 injections/vial; 1 µL injection volume; 280 °C injector temperature, in splitless mode; the 60 °C initial oven temperature was held for 2 min, followed by a 10 °C·min^−1^ ramp up to a 300 °C final temperature, kept for 15 min. The chromatographic column used for the separation of the compounds was an HP-5MS UI (Agilent Technologies) of 30 m length, 0.250 mm diameter and 0.25 µm film. The MS conditions were: temperature of the electron impact source of the mass spectrometer = 230 °C and of the quadrupole = 150 °C; 70 eV ionization energy. Equipment calibration was conducted using test mixture 2 for apolar capillary columns according to Grob (Supelco 86501) and PFTBA tuning standards. Compound identification was carried out using the NIST11 library [78].

### 4.5. In Vitro Antibacterial Activity Assessment

The antibacterial activity was assessed by determining the minimum inhibitory concentration (MIC). The agar dilution method was used, according to CLSI standard M07-11 [79]. An isolated colony of *X. ampelinus* was incubated in TSB liquid medium at 26 °C for 18 h. Starting from a 10^8^ CFU·mL^−1^ concentration, serial dilutions were then conducted to obtain a final inoculum of ~10^4^ CFU·mL^−1^. Subsequently, bacterial suspensions were delivered to the surface of TSA plates amended with the treatments at concentrations ranging from 62.5 to 1500 μg·mL^−1^. The plates were incubated at 26 °C for 24 h. The procedure for *E. amylovora* was identical, except for the incubation temperature (30 °C). MICs were visually determined as the lowest concentrations at which no bacterial growth was visible in the agar dilutions. All experiments were run in triplicate, with each replicate consisting of 3 plates per treatment/concentration.

### 4.6. In Vitro Antifungal Activity Assessment

The antifungal activity of the different treatments was determined according to EUCAST standard antifungal susceptibility testing procedures [80], using the agar dilution method. Aliquots of stock solutions were incorporated onto the PDA medium to obtain concentrations in the 62.5–1500 μg·mL^−1^ range. Mycelial plugs (5 mm in diameter), taken from the margin of 7-day-old *D. seriata* PDA cultures, were transferred to plates amended with aforementioned concentrations of each treatment (3 plates per treatment/concentration, with 2 replicates). Plates were incubated in the dark at 25 °C for 7 days. PDA medium without any amendment was used as the control. Mycelial growth inhibition was estimated according to the formula:

(2) $$\left(\left(d_{c}-d_{t}\right)/d_{c}\right)\times 100,$$

where *d*_c_ and *d_t_* represent the average diameters of the fungal colony of the control and of the treated fungal colony, respectively. EC_50_ and EC_90_ effective concentrations were estimated in IBM SPSS Statistics v.25 (IBM; Armonk, NY, USA) software using PROBIT analysis. The level of interaction was determined according to Wadley’s method [81].

### 4.7. Statistical Analysis

Given that the homogeneity and homoscedasticity requirements were satisfied (according to Shapiro–Wilk and Levene tests, respectively), the mycelial growth inhibition results for *D. seriata* were statistically analyzed in IBM SPSS Statistics v.25 software using one-way analysis of variance (ANOVA), followed by post hoc comparison of means through Tukey’s test at *p* < 0.05.

## 5. Conclusions

A halophyte from the cliffs of the Atlantic coasts of Europe, viz. *Limonium binervosum* (rock sea-lavender) was studied by elemental and thermal analysis, FTIR spectroscopy and GC–MS with a view to its valorization. The use of its biomass as a solid biofuel can be ruled out, given that its higher heating value (in the 16–18 kJ·g^−1^ range) and content of ashes (5.6% and 17% for flowers and leaves, respectively) do not meet the minimum legal requirements, but its high content in fatty acids open the door to potential exploitation as a biofuel feedstock. Another potential application would be related to the use of its hydrometanolic extracts as natural biocontrol products, given that phytochemicals with antimicrobial properties were found in significant amounts: both flower and leaf extracts contained eicosane (4–18%), *β*-sitosterol (9–19%) and tocopherols (7–13%), besides fatty acids and their esters (22% of tetradecanoic acid in the flower extract, and 30% of linolenic and linoleic acids in the leaf extract). The inhibitory activity of the extracts and their main constituents, alone or in combination with chitosan oligomers, was tested in vitro against *X. ampelinus*, *E. amylovora* and *D. seriata* phytopathogens. A remarkable antibacterial activity was observed against *X. ampelinus* (with a MIC value of 250 μg·mL^−1^) and *E. amylovora* (MIC = 500 μg·mL^−1^) for the conjugated complex of the flower extract with COS, which also resulted in an EC_90_ of 914 μg·mL^−1^ against *D. seriata*. In view of these results, the conjugate complexes of this halophyte may be put forward as promising antimicrobial treatments for apple tree and grapevine diseases in organic agriculture.

## Figures and Tables

**Table 1:** Elemental composition (wt%) of *L. binervosum* fractions.

Fraction	C	H	N	S	O	C/N Ratio
Flowers	44.7%	6.5%	1.3%	0.3%	47.2%	34.9
Leaves	40.5%	6.4%	2.6%	0.9%	49.6%	15.7

**Table 2:** Main bands in the FTIR spectra of *L. binervosum* flowers and leaves and their assignments. Peak positions are expressed in cm^−1^.

Fraction		Assignment
Flowers	Leaves	
	3382	Bonded O–H stretching (cellulose)
2921	2918	–CH_2_ asymmetric stretching of alkyls
2852	2850	–CH_2_ symmetric stretching; CH_2_–(C6)– bending (cellulose)
2158		CN stretching
1731	1728	C=O stretching of alkyl ester
1653	1636	Amide I; C=C stretching; C=O stretching
1605	1617	Aromatic C=C skeletal stretching; COO^−^ antisymmetric stretching (polygalacturonic, pectin ester)
1558		Amide II; COO^−^ symmetric stretching; polynuclear aromatics
1515	1517	C=C stretching vibrations of aromatic structures
1457 1441	1462	O–CH_3_ stretching; C–H bending of CH_2_ or CH_3_
	1417	CH_2_ symmetric bending; aromatic C=C; COO symmetric stretching
1362	1372	C–H (cellulose)
1236	1236	Amide III; C–C–O asymmetric stretching acetylated glucomannan; C–O stretching of aryl ether; C–O and OH of COOH groups
1162	1168	C–O–C in bridge asymmetric; C–C in plane
1100	1104	C–O–C symmetric stretching
1017	1021	C–H bending (typical of carotenes); polygalacturonic acid (a variety of pectin in plant cuticles)
	874	*β*-glycosidic linkages (glucose units of cellulose chains)
832	830	O–C=O in-plane deformation or a CH_2_ rocking deformation
720		In-plane bending or rocking of the methylenes (–CH_2_–)
668		C–C out-of-plane bending

**Table 3:** Main compounds identified in *L. binervosum* flower hydromethanolic extract by GC‒MS.

Peak	R_t_ (min)	Area (%)	Assignment	MW (Da)	Qual
2	11.842	0.92	geranyl acetate or 2,6-octadien-1-ol, 3,7-dimethyl-, acetate (stereoisomers)	196.3	90; 86
3	17.154	1.03	bicyclo[3.1.1]heptane, 2,6,6-trimethyl-, (1α,2β,5α) (also named *trans*-pinane)	138.3	90
6	18.405	4.94	tetradecanoic acid	228.4	93
7	19.666	1.07	heneicosane; hexacosane	296.6; 366.7	98; 92
11	21.458	17.61	eicosane; hexadecane, 2,6,10,14-tetramethyl-; heptadecane	282.5; 282.5; 240.5	97; 97; 96
13	23.060	3.36	heneicosane; pentacosane	296.6; 352.7	96; 93
17	24.608	16.87	tetradecanoic acid, 2-hydroxy-, methyl ester (or methyl 2-hydroxy tetradecanoate)	258.4	93
18	25.095	1.66	tetracosane; heptadecane, 9-octyl-; tricosane, 2-methyl-	338.7; 352.7; 338.7	93; 93; 86
19	25.309	1.26	1,2-tetradecanediol	230.4	64
20	25.538	2.35	squalene	410.7	98
21	25.592	1.21	pentacosane, 13-undecyl-; heneicosane, 3-methyl-	507; 310.6	52; 38
22	25.708	0.90	octacosane; hexacosane	394.8; 366.7	99; 98
23	26.025	14.37	1,2-tetradecanediol	230.4	90
28	27.252	1.13	*γ-*tocopherol	416.7	98
29	27.554	1.23	fumaric acid, 3,5-difluorophenyl dodecyl ester; Z-14-nonacosane	396.5; 406.8	68; 64
30	27.607	3.19	octacosyl trifluoroacetate; tetratriacontyl pentafluoropropionate	506.8; 640.9	38; 38
31	27.992	5.56	vitamin E; dl-*α*-tocopherol	430.7; 430.7	99; 99
33	29.112	1.74	campesterol	400.7	62
34	30.173	8.83	*γ*-sitosterol; *β*-sitosterol	414.7; 414.7	99; 95
35	31.166	1.59	2-ethylacridine	207.3	90

Rt: retention time; MW: molecular weight; Qual: percentage of similarity between the molecules present in the sample and those registered in the NIST11 library. When more than one possible assignment is indicated, MW and Qual values for each of the compounds are separated by a semicolon.

**Table 4:** Main compounds identified in *L. binervosum* leaf hydromethanolic extract by GC‒MS.

Peak	R_t_ (min)	Area (%)	Assignment	MW (Da)	Qual
1	17.154	5.41	bicyclo[3.1.1]heptane, 2,6,6-trimethyl-, (1*α*,2*β*,5*α*) (also named (-)-*trans*-pinane); 3-octadecyne	138.3; 250.5	64;58
4	17.593	2.20	cyclohexanol, 1-ethynyl-; phytol, acetate; 1-hexadecyne	124.2; 338.6; 222.4	38; 38; 38
5	18.026	9.83	hexadecanoic acid, methyl ester	270.5	99
6	18.386	4.25	*n*-hexadecanoic acid; *n*-decanoic acid	256.4; 172.3	99; 90
7	19.667	7.63	9,12-octadecadienoic acid (Z,Z)-, methyl ester	294.5	99
8	19.740	22.26	9,12,15-octadecatrienoic acid, methyl ester, (Z,Z,Z)-; 9,12,15-octadecatrienoic acid, (Z,Z,Z)-	292.5; 278.4	99; 95
9	19.832	3.80	phytol	296.5	98
12	25.538	1.08	squalene	410.7	99
13	25.962	2.82	nonacosane; eicosane; docosane	408.8; 282.5; 310.6	99; 98; 96
14	26.415	1.77	*δ*-tocotrienol (or 2H-1-benzopyran-6-ol, 3,4-dihydro-2,8-dimethyl-2-(4,8,12-trimethyltridecyl)-, [2R-[2*(4R*,8R*)]]-)	396.6	98
15	27.125	1.14	*β*-tocopherol	416.7	99
16	27.252	1.84	*γ*-tocopherol; *β*-tocopherol; *δ*-tocopherol, o-methyl-	416.7; 416.7; 416.7	97; 94; 94
17	27.476	1.21	eicosane; octadecane	282.5; 254.5	96; 96
18	27.607	1.57	1H-indole-2-carboxylic acid, 6-(4-ethoxyphenyl)-3-methyl-4-oxo-4,5,6,7-tetrahydro-, isopropyl ester; *n*-methyl-1-adamantaneacetamide	355.4; 207.31	40; 38
19	27.987	8.08	*α*-tocopherol	416.7	99
20	28.070	1.35	phytol, acetate; 2-(4-fluoro-phenyl)-4-(3-methyl-benzylidene)-4h-oxazol-5-one	338.6; 281.3	49; 43
21	30.163	19.15	*γ*-sitosterol; *β*-sitosterol	414.7; 414.7	99; 99

Rt: retention time; MW: molecular weight; Qual: percentage of similarity between the molecules present in the sample and those registered in the NIST11 library. When more than one possible assignment is indicated, MW and Qual values for each of the compounds are separated by a semicolon.

**Table 5:** Antibacterial activity of chitosan oligomers (COS), *L. binervosum* flower and leaf hydromethanolic extracts, their main constituents (eicosane, tetradecanoic acid, linolenic acid, *β*-sitosterol and vitamin E), and their corresponding conjugate complexes (COS–flower extract, COS–leaf extract, COS–eicosane, COS–tetradecanoic acid, COS–linolenic acid, COS–β-sitosterol and COS–vitamin E) against the two phytopathogenic bacteria under study at different concentrations (expressed in μg·mL^−1^).

Pathogen	Compound	Concentration (μg·mL^−1^)									
		62.5	93.75	125	187.5	250	375	500	750	1000	1500
*X. ampelinus*	COS	+	+	+	+	+	+	+	+	+	−
	Flower extract	+	+	+	+	+	+	+	+	+	−
	Leaf extract	+	+	+	+	+	+	+	+	+	−
	Eicosane	+	+	+	+	+	+	+	+	−	−
	*β*-sitosterol	+	+	+	+	+	+	+	+	−	−
	Tetradecanoic acid	+	+	+	+	+	+	−	−	−	−
	Linolenic acid	+	+	+	+	+	+	−	−	−	−
	Vitamin E	+	+	+	+	+	+	−	−	−	−
	COS–flower extract	+	+	+	+	−	−	−	−	−	−
	COS–leaf extract	+	+	+	+	+	+	+	−	−	−
	COS–eicosane	+	+	+	+	+	+	+	−	−	−
	COS–*β*-sitosterol	+	+	+	+	+	+	−	−	−	−
	COS–tetradecanoic acid	+	+	+	+	−	−	−	−	−	−
	COS–linolenic acid	+	+	+	+	−	−	−	−	−	−
	COS–vitamin E	+	+	+	+	−	−	−	−	−	−
*E. amylovora*	COS	+	+	+	+	+	+	+	+	+	−
	Flower extract	+	+	+	+	+	+	+	+	+	−
	Leaf extract	+	+	+	+	+	+	+	+	+	−
	Eicosane	+	+	+	+	+	+	+	+	+	+
	*β*-sitosterol	+	+	+	+	+	+	+	+	+	−
	Tetradecanoic acid	+	+	+	+	+	+	−	−	−	−
	Linolenic acid	+	+	+	+	+	+	−	−	−	−
	Vitamin E	+	+	+	+	+	+	+	−	−	−
	COS–flower extract	+	+	+	+	+	+	−	−	−	−
	COS–leaf extract	+	+	+	+	+	+	+	−	−	−
	COS–eicosane	+	+	+	+	+	+	+	+	+	−
	COS–*β*-sitosterol	+	+	+	+	+	−	−	−	−	−
	COS–tetradecanoic acid	+	+	+	+	−	−	−	−	−	−
	COS–linolenic acid	+	+	+	+	−	−	−	−	−	−
	COS–vitamin E	+	+	+	+	−	−	−	−	−	−

“+” and “−“ indicate presence and absence of bacterial growth, respectively.

**Table 6:** EC_50_ and EC_90_ effective concentrations for the different treatments, expressed in µg·mL^−1^.

EC	COS	Flower Extract	Leaf Extract	Eicosane	*β*-Sitosterol	Tetradecanoic	Linolenic	Vitamin E
EC_50_	744 ± 42	845 ± 19	1033 ± 107	154 ± 29	82 ± 11	153 ± 17	227 ± 17	212 ± 13
EC_90_	1180 ± 46	1555 ± 71	2167 ± 215	1023 ± 96	151 ± 26	394 ± 49	538 ± 73	434 ± 57
**EC**		**COS–Flower Extract**	**COS–Leaf Extract**	**COS–Eicosane**	**COS–*β*-Sitosterol**	**COS–Tetradecanoic**	**COS–Linolenic**	**COS–Vitamin E**
EC_50_		611 ± 33	625 ± 20	234 ± 13	51 ± 2	109 ± 2	39 ± 1	217 ± 7
EC_90_		914 ± 75	966 ± 64	678 ± 54	124 ± 4	130 ± 4	129 ± 8	406 ± 10

**Table 7:** Synergy factors, estimated according to Wadley’s method, for the conjugate complexes under study.

EC	COS–Flower Extract	COS–Leaf Extract	COS–Eicosane	COS–*β*-Sitosterol	COS–Tetradecanoic	COS–Linolenic	COS–Vitamin E
EC_50_	1.30	1.38	1.09	2.90	2.33	8.98	1.52
EC_90_	1.47	1.58	1.63	2.15	4.55	5.75	1.56

## Data Availability

The data presented in this study are available on request from the corresponding author. The data are not publicly available due to their relevance to be part of an ongoing Ph.D. Thesis.

## Supplementary Materials

The following are available online at https://www.mdpi.com/article/10.3390/plants10091852/s1, Figure S1: TG, DTG and DSC curves for *L. binervosum* flowers, Figure S2: TG, DTG and DSC curves for *L. binervosum* leaves, Figure S3: GC–MS spectrum of *L. binervosum* flower hydromethanolic extract, Figure S4: GC–MS spectrum of *L. binervosum* leaf hydromethanolic extract, Figure S5: Growth inhibition of *D. seriata* for the conjugate complexes under study, Table S1: GC‒MS results for the *L. binervosum* flower hydromethanolic extract, Table S2: GC‒MS results for the *L. binervosum* leaf hydromethanolic extract.
