# Supplementary material for: Characterization and Antimicrobial Activity of a Halophyte from the Asturian Coast (Spain): Limonium binervosum (G.E.Sm.) C.E.Salmon
Source: Plants (Basel). 2021 Sep 7;10(9):1852. doi: 10.3390/plants10091852 (PMC8468922; doi:10.3390/plants10091852)
Supplement: Supplementary file 1 [file plants-10-01852-s001.zip › plants-1353175-SI.pdf]

Characterization and antimicrobial activity of a halophyte from the Asturian coast (Spain): *Limonium binervosum* (G.E.Sm.) C.E.Salmon

SUPPORTING INFORMATION

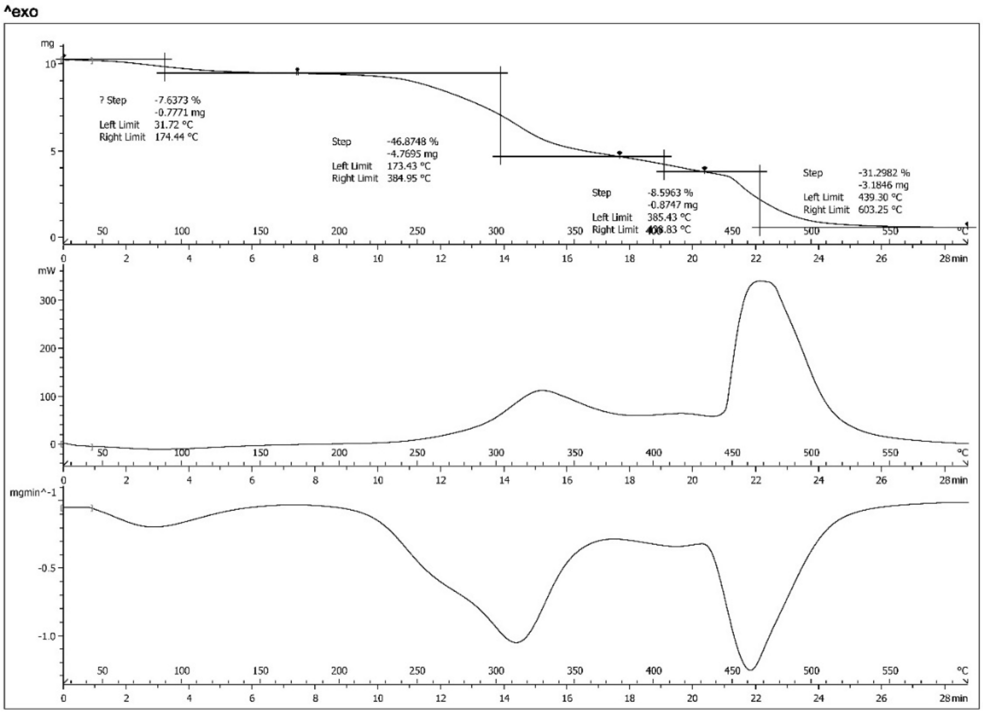

Figure S1. TG (*top*), DTG (*center*) and DSC (*bottom*) curves for *L. binervosum* flowers.

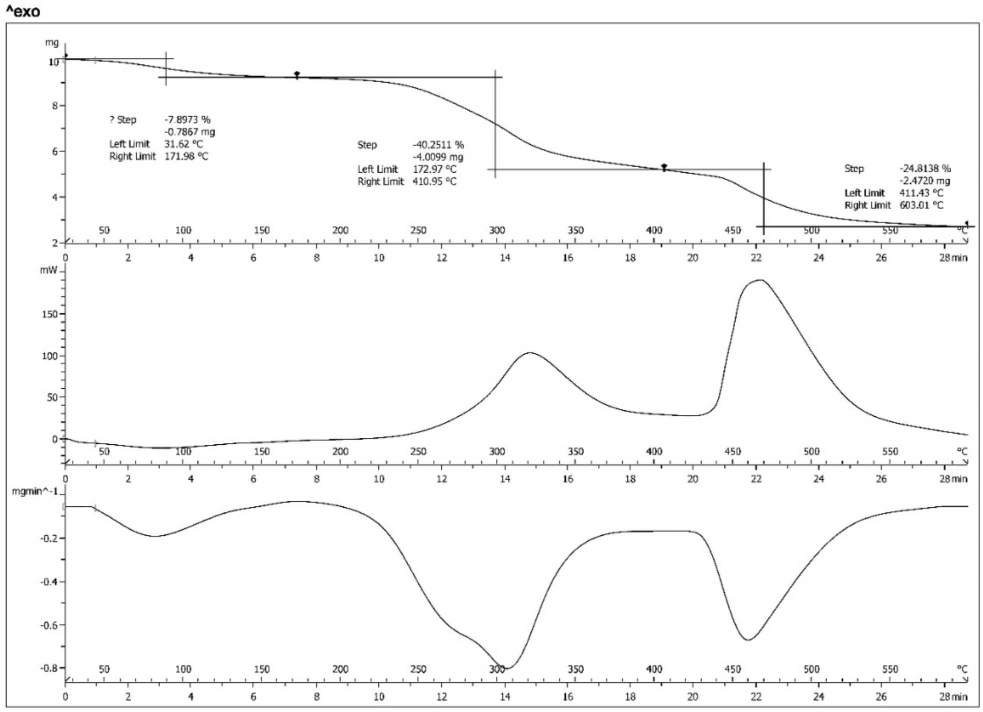

Figure S2. TG (*top*), DTG (*center*) and DSC (*bottom*) curves for *L. binervosum* leaves.

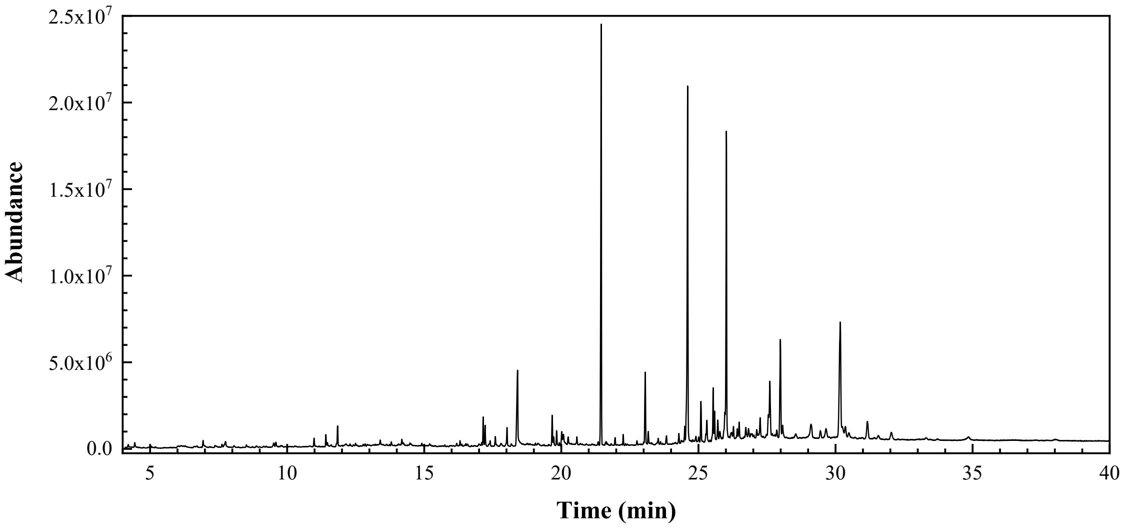

Figure S3. GC–MS chromatogram of *L. binervosum* flower hydromethanolic extract.

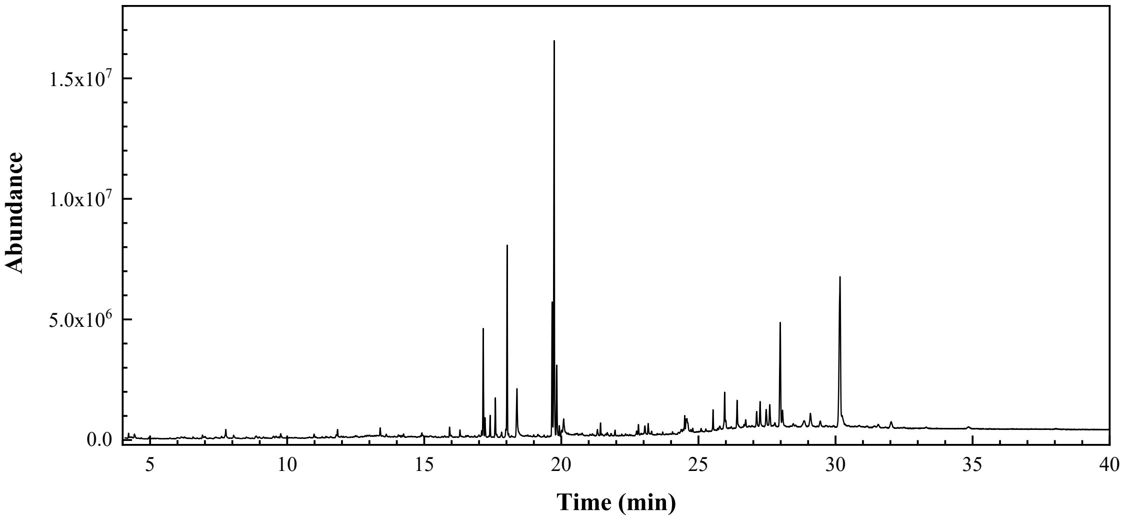

Figure S4. GC–MS chromatogram of *L. binervosum* leaf hydromethanolic extract.

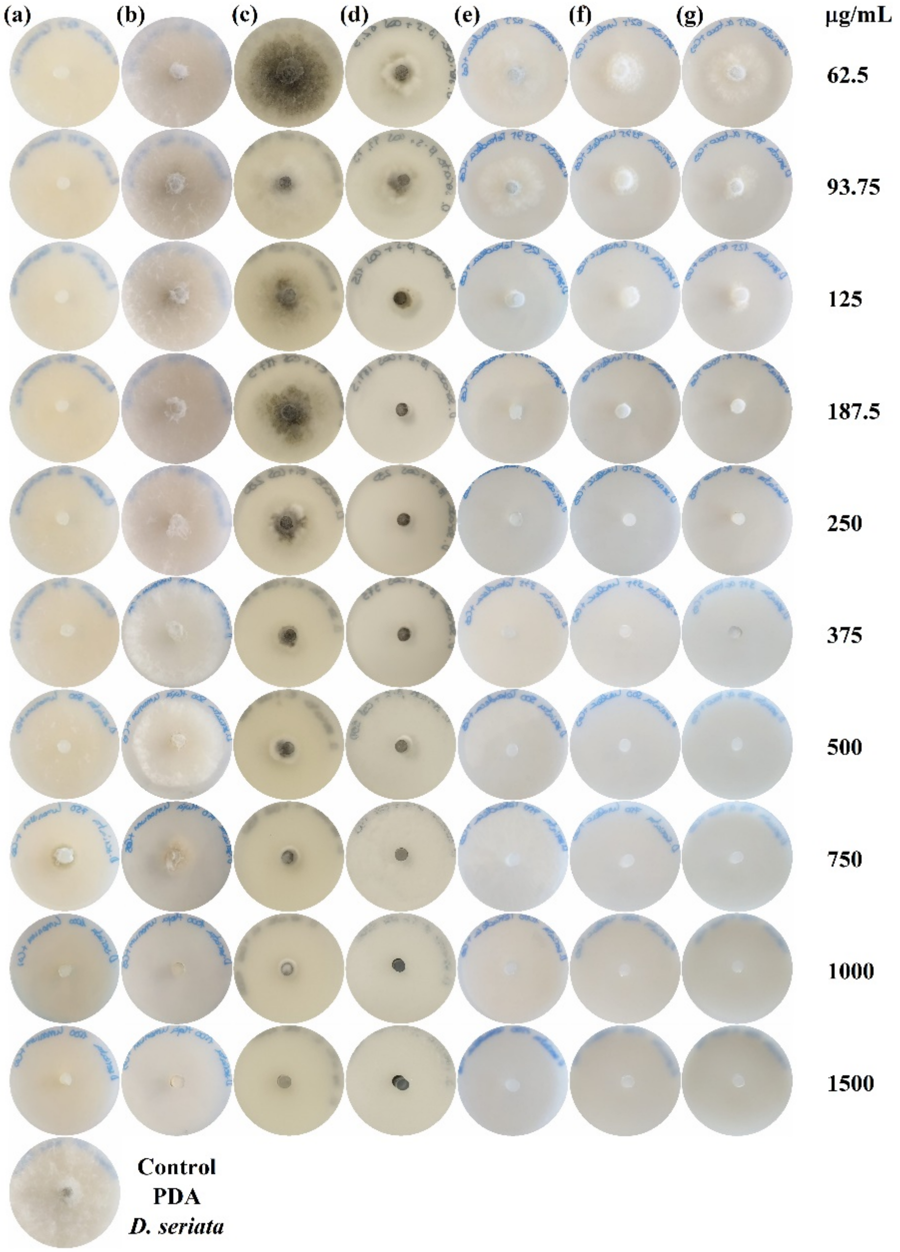

Figure S5. Growth inhibition of *D. seriata* for the conjugate complexes under study: **(a)** COS–flower extract; **(b)** COS–leaf extract; **(c)** COS–eicosane; **(d)** COS–*β*-sitosterol; **(e)** COS–tetradecanoic acid; **(f)** COS–linolenic acid; **(g)** COS–vitamin E. Treatment doses from top to bottom: 62.5, 93.75, 125, 187.5, 250, 375, 500, 750, 1000 and 1500 µg·mL^{-1}. The control (PDA medium without amendments) is shown in the bottom left corner. Only one replicate is shown.

Table S1. GC–MS results for the *L. binervosum* flower hydromethanolic extract.

PK	RT	Area Pct	Library/ID	Ref	CAS	Qual
1	11.4140	0.42	Benzeneethanol, 4-hydroxy-	17329	000501-94-0	72
2	11.8425	0.92	Geranyl acetate	57977	000105-87-3	90
3	17.1542	1.03	Bicyclo[3.1.1]heptane, 2,6,6-trimethyl-, [1R-(1.alpha.,2.beta.,5.alpha.)]-	17016	004795-86-2	90
4	17.2223	0.81	2-Pentadecanone, 6,10,14-trimethyl-	117627	000502-69-2	96
5	18.0208	0.86	Hexadecanoic acid, methyl ester	119400	000112-39-0	99
6	18.4054	4.94	Tetradecanoic acid	84452	000544-63-8	93
7	19.6664	1.07	Heneicosane	141426	000629-94-7	98
8	19.8319	0.57	Phytol	141393	000150-86-7	95
9	20.0169	0.67	9,12-Octadecadienoic acid (Z,Z)-	127649	000060-33-3	99
10	20.0802	0.67	9,12,15-Octadecatrienoic acid, (Z,Z,Z)-	126110	000463-40-1	97
11	21.4580	17.61	Eicosane	129491	000112-95-8	97
12	22.2565	0.48	Tetracosane	175559	000646-31-1	99
13	23.0598	3.36	Heneicosane	141424	000629-94-7	96
14	23.1718	0.51	Glycerol 1-palmitate	169227	000542-44-9	91
15	24.2867	0.39	Tetracosane	175556	000646-31-1	95
16	24.5009	0.60	Quinoline-4-carboxamide, 2-phenyl-N-(3,4-difluorophenyl)-	190680	303133-50-8	49
17	24.6080	16.87	Tetradecanoic acid, 2-hydroxy-, methyl ester	109146	056009-40-6	93
18	25.0949	1.66	Tetracosane	175557	000646-31-1	93
19	25.3091	1.26	1,2-Tetradecanediol	86137	021129-09-9	64
20	25.5379	2.35	Squalene	215927	000111-02-4	98
21	25.5915	1.21	Pentacosane, 13-undecyl-	235975	055517-89-0	52
22	25.7083	0.90	Octacosane	209565	000630-02-4	99
23	26.0248	14.37	1,2-Tetradecanediol	86137	021129-09-9	90
24	26.2780	0.54	Tetracosyl trifluoroacetate	227109	1000351-76-4	46
25	26.4192	0.50	2H-1-Benzopyran-6-ol, 3,4-dihydro-2,8-dimethyl-2-(4,8,12-trimethyltridecyl)-, [2R-[2R*(4R*,8R*)]]-	213180	000119-13-1	99
26	26.4873	0.82	Eicosane, 9-octyl-	209567	013475-77-9	53
27	26.7308	0.51	1-Nonadecene	115906	018435-45-5	83
28	27.2517	1.13	.gamma.-Tocopherol	218064	007616-22-0	98
29	27.5536	1.23	Fumaric acid, 3,5-difluorophenyl dodecyl ester	210152	1000339-37-8	68
30	27.6071	3.19	Octacosyl trifluoroacetate	235928	1000351-74-9	38
31	27.9918	5.56	Vitamin E	222346	000059-02-9	99
32	28.0745	0.84	Cyclohexane, (2,2-dimethylcyclopentyl)-	45742	061142-23-2	68
33	29.1115	1.74	Campesterol	212387	000474-62-4	62
34	30.1729	8.83	.gamma.-Sitosterol	217434	000083-47-6	99
35	31.1661	1.59	9,19-Cyclolanost-24-en-3-ol, (3.beta.)-	221205	000469-38-5	90

Table S2. GC–MS results for the *L. binervosum* leaf hydromethanolic extract.

PK	RT	Area Pct	Library/ID	Ref	CAS	Qual
1	17.1544	5.4114	Bicyclo[3.1.1]heptane, 2,6,6-trimethyl-, (1.alpha.,2.beta.,5.alpha.)-	17013	006876-13-7	64
2	17.2225	1.0098	2-Hexadecene, 3,7,11,15-tetramethyl-, [R-[R*,R*-(E)]]-	127782	014237-73-1	93
3	17.4027	1.1214	9-Octadecyne	102606	035365-59-4	90
4	17.5925	2.1968	Cyclohexanol, 1-ethynyl-	10508	000078-27-3	38
5	18.0258	9.8341	Hexadecanoic acid, methyl ester	119407	000112-39-0	99
6	18.3861	4.2543	n-Hexadecanoic acid	107549	000057-10-3	99
7	19.6666	7.634	9,12-Octadecadienoic acid (Z,Z)-, methyl ester	139724	000112-63-0	99
8	19.7396	22.2609	9,12,15-Octadecatrienoic acid, methyl ester, (Z,Z,Z)-	138095	000301-00-8	99
9	19.8321	3.8008	Phytol	141393	000150-86-7	98
10	20.0853	1.8371	9,12,15-Octadecatrienoic acid, (Z,Z,Z)-	126112	000463-40-1	99
11	24.5011	0.6163	Octocrylene	191340	006197-30-4	83
12	25.5382	1.0831	Squalene	215931	000111-02-4	99
13	25.9617	2.824	Nonacosane	215180	000630-03-5	99
14	26.4145	1.7721	2H-1-Benzopyran-6-ol, 3,4-dihydro-2,8-dimethyl-2-(4,8,12-trimethyltridecyl)-, [2R-[2R*(4R*,8R*)]]-	213180	000119-13-1	98
15	27.1253	1.1363	.beta.-Tocopherol	218061	000148-03-8	99
16	27.2519	1.8386	.gamma.-Tocopherol	218064	007616-22-0	97
17	27.4759	1.2121	Eicosane	129490	000112-95-8	96
18	27.6073	1.572	1H-Indole-2-carboxylic acid, 6-(4-ethoxyphenyl)-3-methyl-4-oxo-4,5,6,7-tetrahydro-, isopropyl ester	187366	1000316-17-5	40
19	27.9871	8.0828	Vitamin E	222351	000059-02-9	99
20	28.0698	1.3531	Phytol, acetate	175494	1000375-01-4	49
21	30.1634	19.1488	.gamma.-Sitosterol	217434	000083-47-6	99
